# Terra Firma–Forme Dermatosis: Clinical Insights, Dermoscopic and Ultraviolet-Induced Fluorescence Dermoscopy Findings—A Case Report and Literature Review

**DOI:** 10.1155/crpe/9349324

**Published:** 2025-07-16

**Authors:** Nina Łabędź, Dorota Sobolewska-Sztychny, Magdalena Sadowska, Klaudia Kubikowska, Katarzyna Korecka, Joanna Narbutt, Aleksandra Lesiak

**Affiliations:** ^1^Department of Dermatology, Pediatric Dermatology and Dermatological Oncology, Medical University of Łódź, Łódź, Poland; ^2^Department of Dermatology, Poznań University of Medical Sciences, Poznań, Poland; ^3^Laboratory of Autoinflammatory, Genetic and Rare Skin Disorders, Medical University of Łódź, Łódź, Poland; ^4^Department of Dermatology and Venereology, Poznań University of Medical Sciences, Poznań, Poland

**Keywords:** dermoscopy, terra firma-forme dermatosis, ultravioletinduced dermoscopy

## Abstract

Terra firma–forme dermatosis (TFFD) is a benign skin condition characterized by persistent brownish, yellowish, or gray–black patches, primarily affecting children and young adults. Diagnosis is typically clinical but can be enhanced using dermoscopy and ultraviolet-induced fluorescence dermoscopy (UVFD). This case report presents three patients diagnosed with TFFD, highlighting the unique UVFD patterns observed and the effective management strategies employed. The findings underscore the importance of recognizing TFFD to alleviate patient concerns and prevent unnecessary interventions.

## 1. Introduction

Terra firma–forme dermatosis (TFFD) is a benign, acquired dermatologic condition that remains underrecognized. Initially described in 1987 by Duncan, Tschen, and Knox [1], the term “terra firma,” meaning “dry land” in Latin, reflects the characteristic appearance of the skin lesions [1]. Clinically, TFFD manifests as brown, yellow, or gray–black macules and plaques, commonly affecting the face, neck, trunk, and ankles [2]. The condition predominantly affects children and young adults [2].

A distinguishing feature of TFFD is the persistence of these lesions despite thorough washing with soap and water. However, complete resolution can be achieved by wiping the affected areas with 70% isopropyl alcohol, a diagnostic hallmark [2]. While the diagnosis is primarily clinical, dermoscopy and ultraviolet-induced fluorescence dermoscopy (UVFD) can serve as useful adjuncts to facilitate early and accurate identification.

We present three cases of TFFD diagnosed in our hospital over a short period, emphasizing the clinical features, diagnostic methods, and effective management strategies.

## 2. Case Reports

### 2.1. Case 1

**Figure 1:**
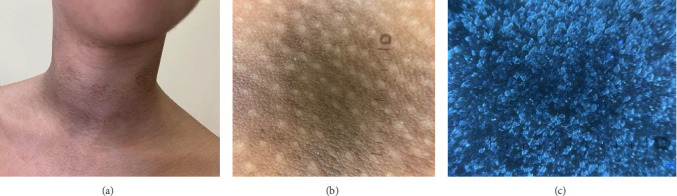
(a) Reticular, brownish plaques on the front and side surfaces of the neck. (b) Dermoscopy shows brownish pigmentation sparing the follicular ostia (DermLite DL5). (c) Bright blue clods and plaques in UVFD (DermLite DL5).

A 15-year-old female with Fitzpatrick skin type IV and a history of alopecia areata presented with asymptomatic, dirt-like hyperpigmented patches on the neck and upper chest, persisting for approximately three months (Figure 1(a)). Despite regular cleansing with soap and water, the lesions remained resistant to removal. The patient reported experiencing bullying at school due to a perceived lack of hygiene. Dermoscopy revealed brownish pigmentation with sparing of the follicular ostia (Figure 1(b)). UVFD demonstrated multiple bluish clods, resembling a “starry sky” pattern. The TFFD plaques appeared sharply demarcated, with their borders often more clearly visible under UVFD than in conventional polarized dermoscopy. Notably, the surrounding unaffected skin did not exhibit fluorescence, which further accentuated the contrast between the lesions and adjacent healthy areas (Figure 1(c)). The characteristic fluorescence seen on UVFD likely reflects the accumulation of keratinocytes and melanin retained in the superficial layers of the epidermis. The lesions were entirely cleared after swabbing with 70% isopropyl alcohol, confirming the diagnosis.

### 2.2. Case 2

**Figure 2:**
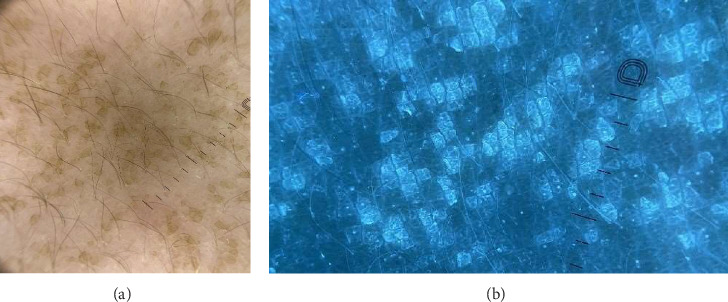
(a) Large polygonal brown clods in dermoscopy (DermLite DL5). (b) Bright blue clods in UVFD (DermLite DL5).

A 17-year-old female presented with an asymmetrical brown area of hyperpigmentation on the back of unknown duration. The lesions were asymptomatic, and the patient reported maintaining regular personal hygiene. Relevant medical history included psoriasis treated with ustekinumab, obesity, and acanthosis nigricans. Physical examination revealed multiple unilateral brown patches with a reticular pattern. Direct microscopic examination of potassium hydroxide (KOH)–prepared specimens was negative for fungal structures. Dermoscopy showed yellowish, large, polygonal clods interspersed with islands of normal skin (Figure 2(a)). UVFD revealed bluish clods of varying shapes, each surrounded by well-demarcated bright rims. Unaffected skin areas did not show fluorescence. In addition, UVFD enhanced the visibility of subtle, subclinical lesions that were poorly perceptible under conventional dermoscopy, allowing for a more accurate assessment of the extent of skin involvement (Figure 2(b)). The lesions were completely removed after rubbing with 70% isopropyl alcohol, confirming the diagnosis of TFFD.

### 2.3. Case 3

**Figure 3:**
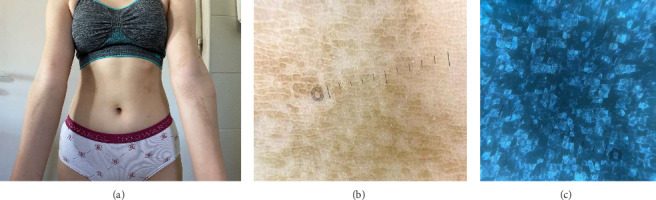
(a) Generalized hyperpigmented lesions on the upper limbs and trunk. (b) Well-delimited, brown clods arranged in a cobblestone pattern in the dermoscopy image (DermLite DL5). (c) Blueish, clear-cut clods in UVFD (DermLite DL5).

A 16-year-old patient was admitted to the department of dermatology with nonpruritic, brown-to-black, dirt-like lesions on the arms, forearms, and abdomen, persisting for approximately seven months (Figure 3(a)). The patient reported experiencing bullying at school due to the appearance of the skin. Attempts to remove the lesions with soap and water were unsuccessful. The patient's medical history was unremarkable. Physical examination revealed symmetrical, brown-black papillomatous plaques. Dermoscopy showed numerous brown, polygonal clods interspersed with areas of normal skin (Figure 3(b)). UVFD revealed bright blue, well-defined clods arranged in a mosaic-like pattern, with sharply demarcated polygonal borders (Figure 3(c)). A clear demarcation was observed between the fluorescent skin lesions, corresponding to the accumulation of melanin, keratin, and external debris, and the unaffected skin, which showed no fluorescence under UVFD. The lesions resolved completely after application of 70% isopropyl alcohol, confirming the diagnosis of TFFD.

## 3. Discussion

TFFD is a rare and underreported dermatologic condition, primarily documented in case reports. The etiology remains uncertain but is hypothesized to involve abnormal keratinocyte maturation, leading to the accumulation of melanin, sebum, and epidermal debris [3]. Exogenous factors such as emollient use, xerosis, and sun exposure may contribute to or exacerbate TFFD, although lesions can also occur in covered areas [4]. Notably, in our cases, lesions were present on both exposed and covered body regions. Some studies suggest a possible role for *Malassezia furfur i*n TFFD development [5]. In addition, TFFD has been reported in postoperative sites, potentially due to reduced skin friction [6].

TFFD often coexists with other dermatologic conditions, particularly atopic dermatitis, and has been associated with psoriasis, acne, vitiligo, seborrheic dermatitis, rosacea, alopecia areata, xerosis, discoid lupus erythematosus, leiomyoma, progressive macular hypomelanosis, and tinea capitis [2, 4, 5]. Clinically, TFFD presents as asymptomatic, hyperpigmented, brownish-to-grayish, hyperkeratotic papules or plaques resembling “dirty skin,” which inspired the name. [2] Commonly affected areas include the neck, face, trunk, navel, ankles, genitals, and skin folds [2, 3].

Diagnosis is often incidental, as seen in our patients, and the lesions can cause significant embarrassment. Although clinical presentation is typically sufficient for diagnosis, adjunct diagnostic tools can enhance accuracy. Wood's lamp examination reveals a chalk-white fluorescence, as described in recent studies. [7, 8] Histopathological examination, though rarely necessary, shows hyperkeratosis, papillomatosis, acanthosis, and basal layer hypermelanosis. [2] In our series, none of the patients required biopsy.

Dermoscopy and UVFD are valuable, noninvasive diagnostic tools. Dermoscopy commonly reveals brown polygonal or hexagonal clods in a mosaic pattern, resembling a “stone pavement” or “cobblestone” appearance. Seborrheic keratosis-like patterns and perifollicular hyperpigmentation have also been reported. [9–12] UVFD, a novel technique using fluorochromes to induce fluorescence, highlights bright blue plaques with distinct patterns. [13].

In our cases, UVFD consistently revealed bluish clods and plaques forming various shapes and patterns, which we termed the “starry sky” and three-dimensional mosaic patterns. UVFD revealed a bright border composed of polygonal, scale-like structures characteristic of TFFD. The fluorescence observed under UVFD may correspond to the retention of keratin and melanin within the epidermis. Similar to dermoscopy, UVFD highlights affected areas with luminous clods separated by nonluminous, unaffected skin.

The differential diagnosis includes dermatosis neglecta, acanthosis nigricans, dirty neck syndrome of atopic dermatitis, ichthyosis, confluent and reticulated papillomatosis, and pityriasis versicolor (Table 1) [2]. The definitive diagnostic and therapeutic test is the alcohol swab test, also known as the Skin Modified by Alcohol Rubbing Test (SMART) [18]. Lesions are effectively removed by rubbing with 70% isopropyl alcohol, whereas washing with soap and water is ineffective.

Treatment options described in the literature include topical calcineurin inhibitors, salicylic acid, alpha hydroxy acids, topical retinoids, and fractional CO_2_ laser [2, 19].

## 4. Conclusions

TFFD is a benign skin condition that, although clinically harmless, can cause significant cosmetic and psychological distress. Prompt diagnosis is achievable through noninvasive techniques such as dermoscopy and UVFD, thereby avoiding unnecessary laboratory investigations or skin biopsies. Treatment with 70% isopropyl alcohol is simple and highly effective, leading to complete resolution of lesions. We emphasize the importance of awareness among healthcare professionals, particularly dermatologists and pediatricians, to recognize and diagnose this often overlooked yet relatively common condition, facilitating timely management and alleviation of patient concerns.

## Figures and Tables

**Table 1:** Differential diagnosis of terra firma–forme dermatosis.

Condition	Clinical characteristics	Dermoscopy	Ultraviolet-induced fluorescence dermoscopy
Dermatosis neglecta [14]	Asymptomatic localized, hyperpigmented brownish papules and polygonal plaques, easily removed by simple scrubbing with soap water or alcohol	Irregularly distributed cornflake-like dark brown scales	No data
Acanthosis nigricans [14]	Velvety hyperpigmented patches, usually in skin folds (back of the neck, axilla, and groin), often associated with obesity, diabetes, and insulin resistance	Linear crista cutis and sulcus cutis with brown-to-dark dots in the crista cutis	Our observations: Multiple greenish–blue linear patterns along the crista cutis
Dirty neck syndrome of atopic dermatitis [15]	Reticulate hyperpigmentation of the anterior or anterolateral part of the neck, usually in adults with long-lasting, severe atopic dermatitis; often resistant to treatment	No data	No data
Confluent and reticulated papillomatosis (Gougerot–Carteaud syndrome) [14]	Asymptomatic hyperpigmented papules and/or plaques, with a peripheral, net-like pattern; usually located on the upper trunk and neck of teenagers and young adults	Poorly defined brownish, homogeneous polygonal globules with fine whitish scales, arranged in a cobblestone or “sulci and gyri” pattern	No data
Ichtyosis [16]	Dry, rough skin with fine, whitish or light-brown scales, primarily affecting the extensor surfaces of the upper and lower extremities	Criss-cross pattern of fine white scales; brown rhomboid structures with space in between	No data
Pityriasis versicolor [14, 17]	Scaly hypopigmented or hyperpigmented patches, usually on the upper trunk, neck, and upper arms	Well-defined depigmented (hypopigmented PV) or red-brownish (hyperpigmented PV) structureless areas covered with scales	Light greenish structureless areas in hypopigmented lesions; feature dark greenish structureless areas in hyperpigmented lesions

## Data Availability

The data that support the findings of this study are available from the corresponding author upon reasonable request.
